# The Severity of Dependence Scale detects medication misuse and dependence among hospitalized older patients

**DOI:** 10.1186/s12877-019-1182-3

**Published:** 2019-06-24

**Authors:** Socheat Cheng, Tahreem Ghazal Siddiqui, Michael Gossop, Espen Saxhaug Kristoffersen, Christofer Lundqvist

**Affiliations:** 1 Health Services Research Unit (HØKH), Akershus Univeristy Hospital, PO Box 1000, 1478 Lorenskog, Norway; 2 Institute of Clinical Medicine, Campus Ahus, Faculty of Medicine, University of Oslo, PO Box 1000, 1478 Lorenskog, Norway; 3 Department of Neurology, Akershus Univeristy Hospital, PO Box 1000, 1478 Lorenskog, Norway; 4 Department of General Practice, Institute of Health and Society, University of Oslo, 1130 Blindern, 0318 Oslo, Norway; 5 National Addiction Centre, Institute of Psychiatry, Psychology and Neuroscience, King’s College London, PO Box 48, 4 Windsor Walk, London, SE5 8AF UK

**Keywords:** Medication dependence, SDS, Diagnostic accuracy, Validity and reliability, Old age

## Abstract

**Background:**

In older patients, timely recognition and treatment of medication misuse and dependence are crucial to secure medication safety and to avoid increasing health expenditure. Nonetheless, the detection of this condition remains challenging due to the paucity of screening instruments validated for older people.

This study assesses diagnostic accuracy, reliability, validity and the factor structure of the Severity of Dependence Scale (SDS) in detecting medication misuse and dependence among hospitalized older patients, focusing on prescribed central nervous system depressants (CNSDs): opioid analgesics, benzodiazepines and z-hypnotics.

**Methods:**

246 adults aged 65–90 were recruited consecutively from somatic departments of the Akershus University Hospital, Norway. Among these, 100 patients were identified as prolonged users of CNSDs. Diagnostic accuracy and validity of the SDS were assessed using DSM-IV criteria for substance abuse and dependence as the reference standard. We also performed an exploratory factor analysis and assessment of internal consistency using Cronbach’s alpha.

**Results:**

The area under the ROC curve was 0.86 (95%CI = 0.79–0.93; *p* < 0.001). A score of 5.5 was determined as the optimal cutoff for detecting CNSD misuse and dependence among older patients. Cronbach’s alpha obtained was satisfactory (α = 0.73). There was a significant positive correlation between the SDS score and DSM-IV criteria for substance abuse and dependence (Pearson’s correlation coefficient = 0.61, *p* < 0.001). The uni-dimensionality of the SDS was documented.

**Conclusions:**

The SDS is reliable, valid and capable of detecting medication misuse and dependence among hospitalized older patients, with good diagnostic performance. The scale thus holds promise for use in both clinical and research contexts.

**Trial registration:**

ClinicalTrials.gov Identifier: NCT03162081. Registered 3 May 2017.

**Electronic supplementary material:**

The online version of this article (10.1186/s12877-019-1182-3) contains supplementary material, which is available to authorized users.

## Background

Central nervous system depressants (CNSDs) such as sedatives and opioid analgesics are commonly prescribed medications for the treatment of chronic pain, insomnia and anxiety in old age. There is still limited evidence on long-term efficacy, though a growing amount of literature accentuates abuse potential and serious adverse effects of these medications [1, 2]. This underlines the importance of rational use and prescription of CNSDs for older patients. To achieve this, one of the important steps is for clinicians to be able to timely recognize older patients at risk or suffering from medication misuse and dependence.

Medication misuse and dependence in older patients is a condition characterized by persistent and compulsive use of a medication despite impairment in physical, social and psychological health, occurring in patients aged 65 and older. Though misuse of prescribed medications may be seen as somewhat different from illegal substance and alcohol misuse, there has been a growing concern about the consequences of such misuse in older people. Given the vulnerability to adverse drug events, timely recognition and treatment of such misuse and dependence are important to secure medication safety, and to avoid long-term consequences and increasing health expenditure [3]. Nonetheless, the detection of this condition remains challenging, partly due to the paucity of screening instruments validated in older people [4].

Abuse and dependence on substances are diagnosable, as demonstrated in younger cohorts. With advances in medical and addiction sciences, many instruments have been tested for their accuracy in measuring dependence. However, as most of the instruments are not specifically designed for elders, there is no single and widely accepted tool for detecting medication misuse and dependence among geriatric patients [4].

The Severity of Dependence Scale (SDS) is commonly used for assessing severity of dependence among substance users. The strength of this scale is that it is brief, user-friendly and time-efficient [5]. However, it remains to be established whether the scale can accurately identify presence and absence of the aforementioned condition in older subjects. Moreover, taking into account age-related changes in cognitive function [6], exploring how item responses correlate and explain the construct validity of the scale is of importance.

This study assesses diagnostic accuracy, reliability, validity of the SDS in detecting medication misuse and dependence among hospitalized older patients as defined by the Diagnostic and Statistical Manual of Mental disorders, Fourth Edition (DSM-IV) [7], focusing on three commonly prescribed CNSDs: opioid analgesics, benzodiazepines and z-hypnotics. In addition, we explored the factor structure of the scale in this population.

## Method

### Study design

The study was cross-sectional. Data collection was planned and piloted before the performance of both the index test and reference standard. Ethical approval was obtained from both the Regional Committee for Medical Research Ethics – South East Norway and the Data Protection Office at Akershus University Hospital. All participants provided written informed consents.

### Participants

We consecutively recruited 246 older patients between May 2017 and August 2018 from three somatic departments of the Akershus University Hospital, Norway: Geriatrics, General Medicine and Neurology. From this group, we identified 100 patients aged 65–90 that had prolonged use (≥ four weeks) of benzodiazepines, opioid analgesics and/or z-hypnotics. This was done through structured interviews and reviewing medical records. We excluded those presenting with one of the following conditions: Mini-Mental State Examination (MMSE) score ≤ 21 [8, 9], pre-existing severe depression, stroke, dementia, psychotic disorders, serious visual or hearing impairment, precluding participation, palliative treatment, insufficient Norwegian language, and lack of written informed consent.

### Test methods

Prior to the study, assessors were trained in delivery and interpretation of the test results for both the index test and reference standard by an advisory board including neuropsychologists and senior researchers.

The SDS (index test) is a five-item questionnaire for measuring psychological components of dependence. The scale has been proved to be valid and reliable in the general population, across substances and settings [5, 10]. In this study, eligible participants were asked to respond to the items of the SDS with four options scoring from 0 to 3, yielding a total score of 0–15. The higher the total score, the higher the degree of dependence (Additional file 7). Execution time was roughly one minute.

The DSM-IV (reference standard) is one of the most widely used tools for diagnosing substance-related disorders. The usability of DSM-IV criteria for substance abuse and dependence is well-documented in the general population. The strength of the manual derives from its multiaxial approach [7, 11]. In addition, there are well established structured diagnostic interview guides such as the Mini International Neuropsychiatric Interview (MINI) [12] that may be used in order to arrive at the DSM-IV criteria-defined diagnoses. We assessed the DSM-IV criteria using the MINI interview right after the SDS without time delay. Dependence was defined to be present if patients met three or more of the dependence criteria. Misuse was confirmed if the dependence criteria were not met and the respondents satisfied one or more of abuse criteria (Additional file 8). A positive outcome on the reference standard was defined as a diagnosis of either substance abuse or dependence. The time needed for the completion of the DSM-IV assessment with the MINI interview ranged from 7 to 10 min.

Both the SDS and the DSM-IV assessment were performed within the first few days of admission and separately for each of the major CNSDs used. No pre-existing information on clinical manifestations or severity of medication misuse and dependence was collected prior to the performance of the index test and reference standard. Both SDS and DSM-IV are non-invasive tests, and no adverse events or negative emotional reactions were observed.

### Analysis

The sample size for assessing diagnostic accuracy of the SDS was estimated by an expected value of the area under the curve (AUC) of 0.85. Using MedCalc statistical software version 18 [13], with a ratio of negative to positive cases of 1, null hypothesis value of 0.5, a significance level of 5% and a power of 80%, the study required 18 cases (9 negatives, 9 positives). The suitability of the data for factor analysis was determined based on the value of Kaiser-Meyer-Olkin Measure (KMO value ≥0.6) and Bartlett’s Test of Sphericity (BTS: *p* < 0.05) [14].

Data analyses were performed in two phases. First, we analyzed data for CNSDs, where all medication groups were included, and thereafter separately for each group of the medications.

Analytical frameworks comprised four parts. Part I dealt with assessing the diagnostic accuracy and optimal cutoff for the SDS for detecting medication misuse and dependence. This involved the use of the receiver operating characteristic (ROC) curve, cross tabulation, Youden’s index, Euclidean distance, and accuracy indices [15]. Part II concerned examining the reliability of the SDS based on internal consistency, using Cronbach’s alpha coefficients. In part III, we assessed convergent validity of the SDS by means of Pearson correlation coefficient. Part IV involved identifying factor structure of the SDS. An exploratory factor analysis was performed on Spearman correlations among items. Principal axis factoring was used to extract initial factors. These factors were then rotated with an oblique rotation method (direct oblimin) to achieve a simple structure of the scale [14]. There was no missing data for either the index test or the reference standard. SPSS software version 25 was used for all statistical procedures.

## Results

### Characteristics of participants

**Fig. 1:**
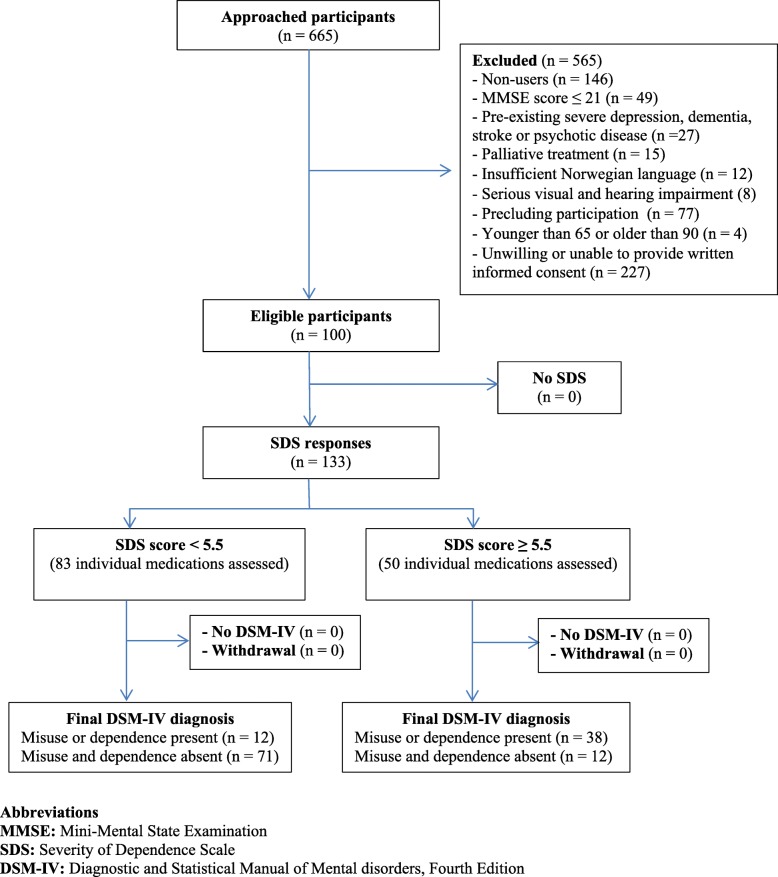
Flow of participants through the study

The flow of participants through the study is shown in Fig. 1. 100 out of 246 patients aged 65–90 (34 men, 66 women) included in the study were identified as prolonged users of opioid analgesics, benzodiazepines or z-hypnotics. Among these users' responses, 38% (*n* = 50) met DSM-IV criteria for substance abuse or dependence. Most were women (68%, *n* = 34) and aged 75–84 (40%, *n* = 20). SDS score for CNSDs had a mean = 4.18 (SD = 3.25); median = 4.00 (range 0–12). The proportion of individual and concomitant use, mean and median of the SDS score for each medication group, and demographic characteristics of CNSD users are presented in Table 1.

**Table 1:** Characteristics, medication use patterns and severity of dependence score of CNSD users

CHARACTERISTICS OF CNSD USERS (*n* = 100)	
Gender	
Men	34 (34%)
Women	66 (66%)
Age, year	
Mean (SD)	78.5 (6.5)
Median (range)	79 (66–90)
Age groups	
65–74	28 (28%)
75–84	51 (51%)
≥ 85	21 (21%)
FREQUENCY OF MEDICATION USE (non-mutually exclusive)	
Benzodiazepines	20 (15%)
Opioid analgesics	45 (34%)
Z-hypnotics	68 (51%)
USE PATTERNS	
Benzodiazepines	7 (7%)
Opioid analgesics	21 (21%)
Z-hypnotics	42 (42%)
Opioid analgesics + Benzodiazepine	4 (4%)
Opioid analgesics + Z-hypnotics	17 (17%)
Benzodiazepines + Z-hypnotics + Opioid analgesics	2 (2%)
SDS SCORE Mean (SD) / Median (range)	
CNSDs	4.2 (3.3) / 4.0 (0–12)
Benzodiazepines	3.5 (3.1) / 3.5 (0–10)
Opioid analgesics	4.1 (3.3) / 3.0 (0–12)
Z-hypnotics	4.4 (3.3) / 4.0 (0–12)

### Test results

#### Diagnostic accuracy

**Fig. 2:**
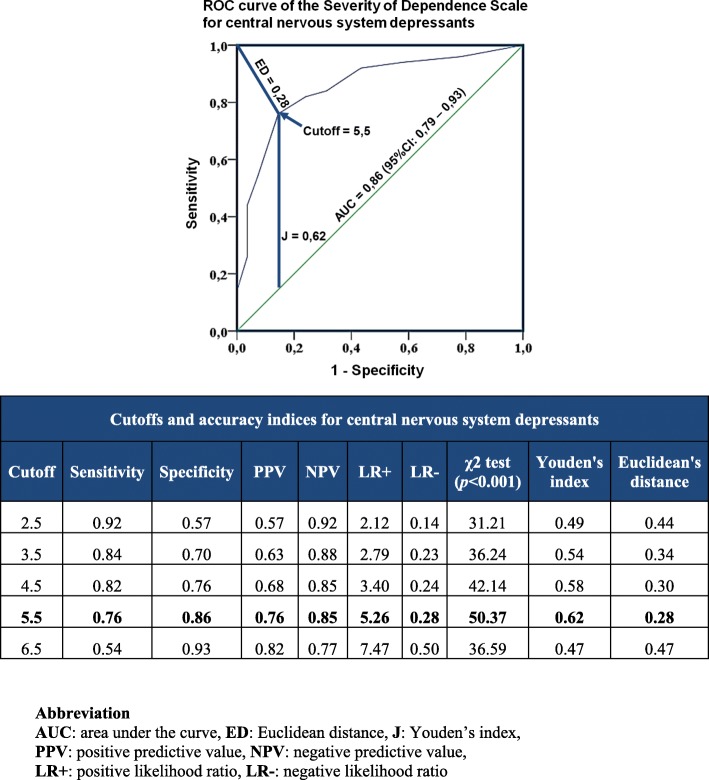
Discriminative ability, cutoffs and accuracy indices of the Severity of Dependence Scale for central nervous system depressants. ROC curve analysis yielded an AUC of 0.86. A score of 5.5 was determined as the optimal cutoff as it generated the best accuracy indices, highest chi-square test value, maximum Youden’s index and minimum Euclidean distance

For CNSDs, the ROC curve analysis yielded an AUC of 0.86 (95%CI = 0.79–0.93; *p* < 0.001). The AUC for benzodiazepines, opioid analgesics and z-hypnotics were 0.89 (95%CI = 0.74–1.00; *p* = 0.004), 0.85 (95%CI = 0.71–0.99; *p* < 0.001) and 0.86 (95%CI = 0.76–0.95; *p* < 0.001) respectively. This suggests that the SDS has high ability to distinguish between older patients with and without CNSD misuse and dependence. Figure 2 shows discriminative ability, cutoffs and accuracy indices of the SDS for CNSDs. ROC curves for each group of medications are available in Additional file 1.

A score of 5.5 was determined as the optimal cutoff for detecting CNSD misuse and dependence among older patients as it generated the best accuracy indices, highest chi-square test value, maximum Youden’s index and minimum Euclidean distance [15, 16] (Fig. 2). This score was also the best cutoff for benzodiazepines and z-hypnotics misuse and dependence. Opioid analgesics had a slightly lower optimal threshold, at 4.5. Optimal cutoffs and accuracy indices for each group of medications are shown in Additional file 2. There were no significant differences in diagnostic accuracy between patients recruited from the three departments.

#### Internal consistency

**Fig. 3:**
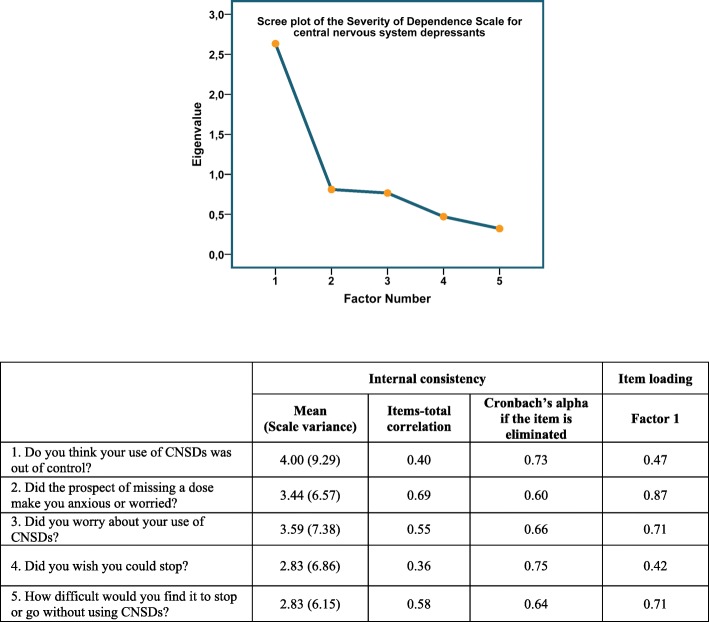
Internal consistency and factor structure of the Severity of Dependence Scale for central nervous system depressants. Evidence of acceptable internal consistency of the scale was found according to the Cronbach’s alpha coefficient (α = 0.73). A single-factor solution was extracted. The factor matrix showed that all items loaded strongly on this factor, with correlation coefficients ranging from 0.42 to 0.87. Scree plot test indicated clear debris after the first factor. These results underline the uni-dimensionality of the SDS in measuring psychological dependence of CNSDs in older patients

Evidence of acceptable internal consistency of the SDS for CNSDs was found according to the Cronbach’s alpha coefficient (α = 0.73) [17]. Removing each of the five items did not significantly increase the value of alpha (Fig. 3).

In line with these findings, Cronbach’s alpha of the SDS for each medication group was also acceptable: benzodiazepines (α = 0.76); opioid analgesics (α = 0.73); z-hypnotics (α = 0.71). Additional files 4, 5 and 6 elaborate results on reliability of the SDS for each group of medications.

#### Convergent validity

Convergent validity of the SDS for CNSDs was established through the significant positive correlation between the SDS score and DSM-IV criteria for substance abuse and dependence (Pearson’s correlation coefficient = 0.61, *p* < 0.001). In the subgroup analysis, the validity was also documented across the three groups of medications: benzodiazepines (Pearson’s correlation coefficient = 0.65 (*p* = 0.002); opioid analgesics (Pearson’s correlation coefficient = 0.62, *p* = 0.001); and z-hypnotics (Pearson’s correlation coefficient = 0.61, *p* < 0.001).

#### Factor structure

KMO test of sampling adequacy was 0.77 and the value of BTS was highly significant (*p* < 0.001). Most correlation coefficients in the Correlation Matrix had the values of ≥0.3. This confirms the suitability of our dataset for factor analysis.

Using Kaiser’s criterion with an eigenvalue of ≥1, a single-factor solution was extracted with the first factor accounting for 53% of the variance. The factor matrix showed that all items loaded strongly on this factor, with correlation coefficients ranging from 0.42 to 0.87. Scree plot test indicated clear debris after the first factor (Fig. 3). These results underline the uni-dimensionality of the SDS in measuring psychological dependence of CNSDs in older patients.

Datasets for each type of medications were also factorable, with KMO test values from 0.61 to 0.78 and high significant values of BTS (*p* < 0.001). Uni-dimensionality was once again documented in the SDS for opioid analgesics and z-hypnotics. The SDS for benzodiazepines, however, had a two-factor solution that explained the maximum amount of the variance. More details are given in the Additional files 3-6.

## Discussion

Overall, our findings show that the SDS has high diagnostic ability to distinguish between hospitalized older patients with and without CNSD misuse and dependence. High accuracy and reliability were documented across the three most common types of CNSDs in our hospital-based sample, at slightly different cutoffs (benzodiazepines and z-hypnotics 5.5; opioid analgesics 4.5). The scale has both construct and concurrent validity. These findings are consistent with those explored in the younger subjects and suggest that the SDS is a valid and reliable tool for detecting the presence of medication misuse and dependence among hospitalized older patients [18–20].

Our study focuses on medication use in a clinical sample of older patients admitted to somatic wards of a general hospital for a variety of reasons and as such should be representative for hospital populations of older people in Norway. We have adhered strictly to the STARD guidelines [21], ethical standards [22], and outcomes were predefined (NCT03162081). We assessed the three most common types of CNSDs using validated and generally accepted criteria for substance abuse and dependence according to the DSM-IV [7, 11].

The use of a hospital-based sample might represent a limitation regarding generalizability of the study findings to the general population. Nonetheless, in this situation random sampling is difficult to achieve as we do not know in advance who the prolonged users of CNSDs are without obtaining informed consents, interviews and access to medical records. In addition, focusing on a hospital population in this way may yield important information on which to base future interventions for a highly vulnerable group. Another issue which suggests some care in interpretation of our results is the relatively high number of non-consenters. It may be that they represent either those with the most serious medical conditions or those that were not interested in being queried regarding their medication use, thus suggesting that our sample may be somewhat biased towards milder cases. Another limitation derives from the lack of test-retest reliability assessment of the SDS. However, behavior may change due to assessment effects and it is also somewhat unclear to what extent the SDS represents a “trait” or a “state” marker which together with recall bias makes a suitable time delay between test and retest difficult to ascertain. It is possible that SDS score may be affected by other factors such as disease severity and comorbidity, though this is not the focus here. Further studies of associated factors are made possible by including the use of the SDS instrument.

As demonstrated, SDS is a straightforward, valid and reliable tool with high capability to confirm and rule out the presence of medication misuse and dependence. The scale could function as a screening and clinical decision supporting tool for recognition and treatment of these entities. Furthermore, we also emphasize that while optimal cutoff for opioid analgesics seems slightly lower, in our sample the dominating medication group was Z-hypnotics and most of opioid users also used these. For this reason and for simplicity of use, we recommend an overall cutoff of 5.5 for all CNSDs among hospitalized older patients.

The scale may also be used for case-finding for future interventions. In primary healthcare research, SDS has already been used as part of interventions to address substance use disorders. Kristoffersen et al. (2014), using the SDS as an identifying tool, demonstrated feasibility and effectiveness of a brief intervention for medication-overuse headache. The scale was found feasible and acceptable for both clinicians and patients within everyday clinical work [23, 24]. Similarly, Copeland et al. (2017) employed the SDS as part of an internet-based brief intervention for cannabis use disorders. The scale was also used as an indicator for assessing changes in the severity of cannabis dependence [25]. Thus, SDS could play two vital roles: to serve as a key component and an outcome variable in the assessment of effectiveness of interventions pertaining to medication misuse and dependence.

Much remains to be discovered. Knowledge of disease profile and burden of medication misuse on older patients and healthcare systems is insufficient for enabling general practitioners and public health officials to develop and implement evidence-based strategies for early detection, prevention and treatment [26]. The SDS could assist future research in quantifying disease occurrence, identifying characteristics and predicting risk factors as well as consequences of medication misuse and dependence.

## Conclusions

Our findings suggest that the Severity Dependence Scale is reliable, valid and capable of detecting medication misuse and dependence among hospitalized older patients as defined by the DSM-IV, with good diagnostic performance. The scale thus holds promise for use in both clinical and research contexts and may be useful both as basis for intervention and to further characterize medication misuse and dependence among older patients.

## Additional files

Additional file 1: ROC curves of the Severity of Dependence Scale for each type of medications. (DOCX 196 kb)

Additional file 2: Cutoff values and accuracy indices of the Severity of Dependence Scale for benzodiazepines, opioid analgesics and z-hypnotics. (DOCX 15 kb)

Additional file 3: Scree plots of the Severity of Dependence Scale for each group of medications. (DOCX 189 kb)

Additional file 4: Internal consistency and item loadings of the Severity of Dependence Scale for benzodiazepines. (DOCX 14 kb)

Additional file 5: Internal consistency and item loadings of the Severity of Dependence Scale for opioid analgesics. (DOCX 14 kb)

Additional file 6: Internal consistency and item loadings of the Severity of Dependence Scale for z-hypnotics. (DOCX 17 kb)

Additional file 7: Severity of dependence scale (SDS). (DOCX 14 kb)

Additional file 8: Mini international neuropsychiatric interview (DSM-IV criteria, Version 6.0.0). (DOCX 26 kb)

## Data Availability

The datasets generated and/or analyzed during the current study are not publicly available due to threats to subject privacy but are available from the corresponding author on reasonable request.

## Abbreviations

**AUC**: Area under the curve
CNSDs: Central nervous system depressants
DSM-IV: Diagnostic and Statistical Manual of Mental disorders (Fourth Edition)
ED: Euclidean distance
J: Youden’s index
NPV: Negative predictive value
PPV: Positive predictive value
SDS: Severity of Dependence Scale
LR +: Positive likelihood ratio
LR-: Negative likelihood ratio
