# Supplementary figures and images for: The Severity of Dependence Scale detects medication misuse and dependence among hospitalized older patients
Source: BMC Geriatr. 2019 Jun 24;19:174. doi: 10.1186/s12877-019-1182-3 (PMC6591833; doi:10.1186/s12877-019-1182-3)

**Additional file 1** ROC curves of the Severity of Dependence Scale for each type of medications

| 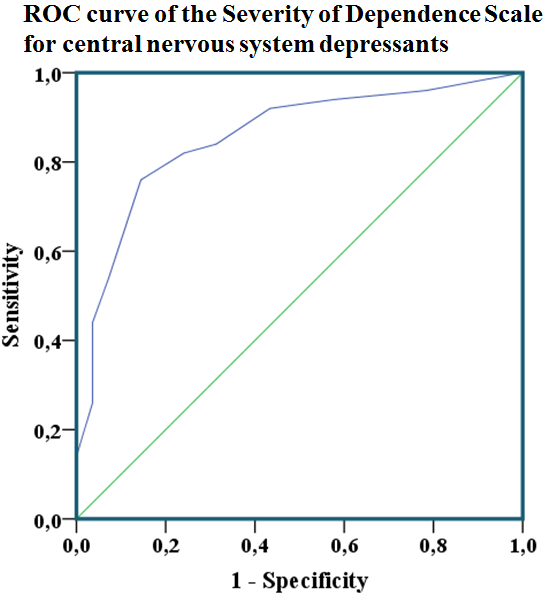 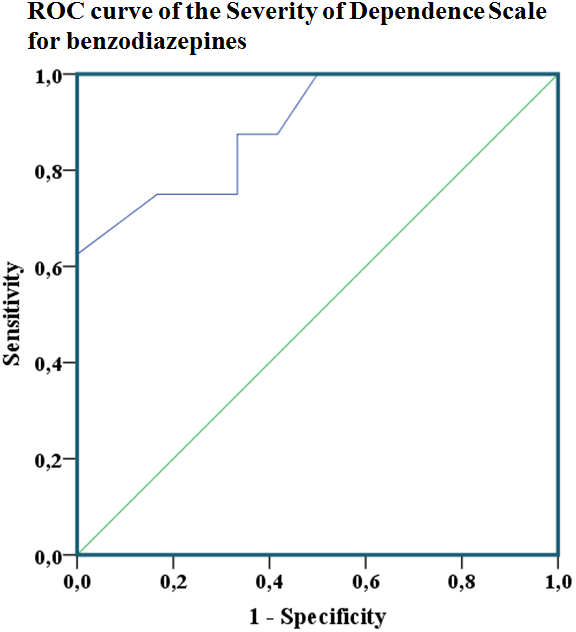  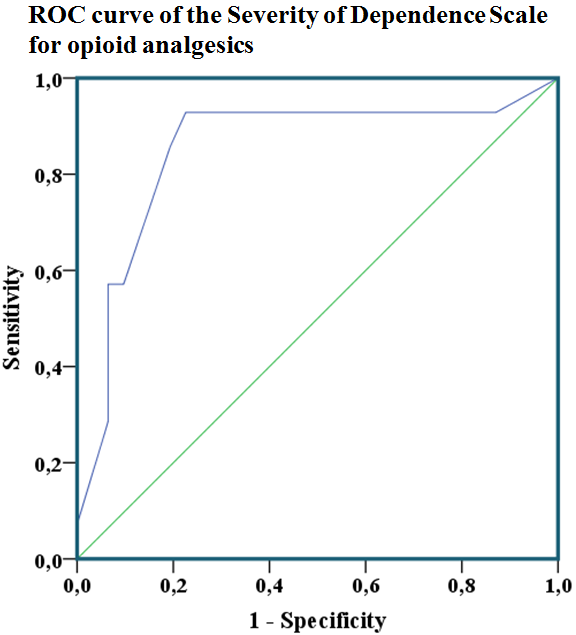 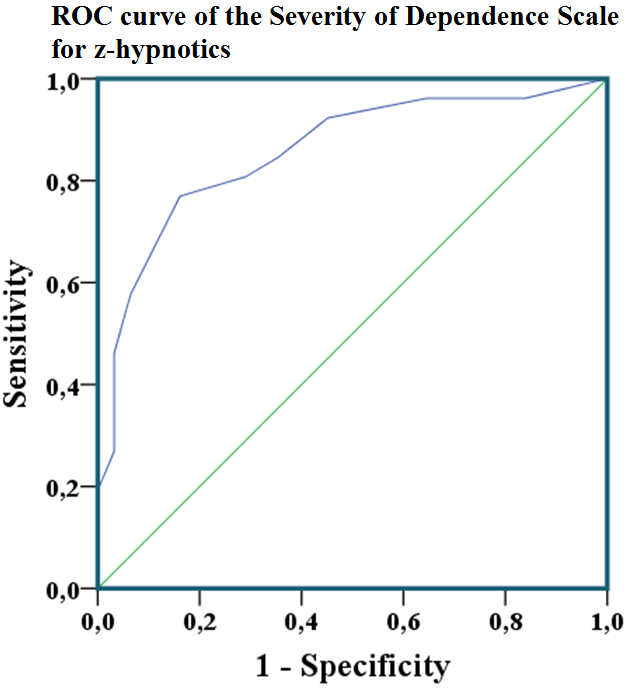 |
| --- |

Supplement: Supplementary file 1 — ROC curves of the Severity of Dependence Scale for each type of medications. (DOCX 196 kb) [file 12877_2019_1182_MOESM1_ESM.docx]

**Additional file 3** Scree plots of the Severity of Dependence Scale for each group of medications


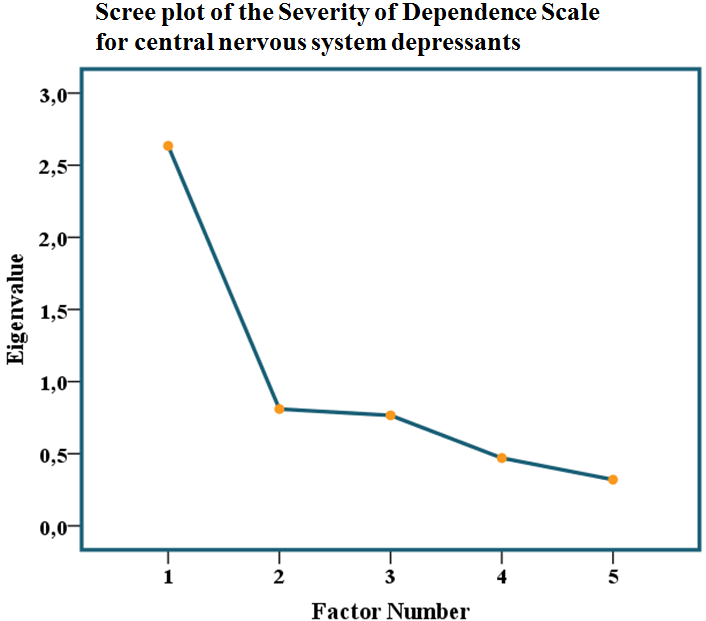

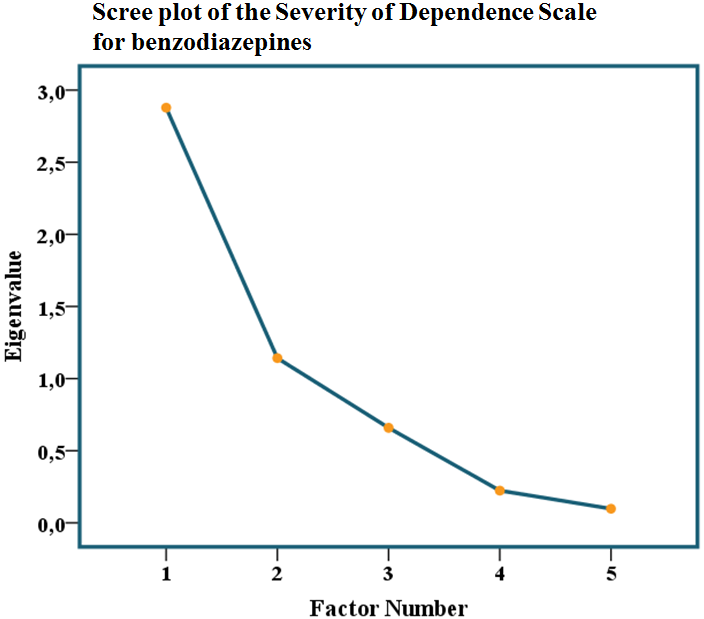


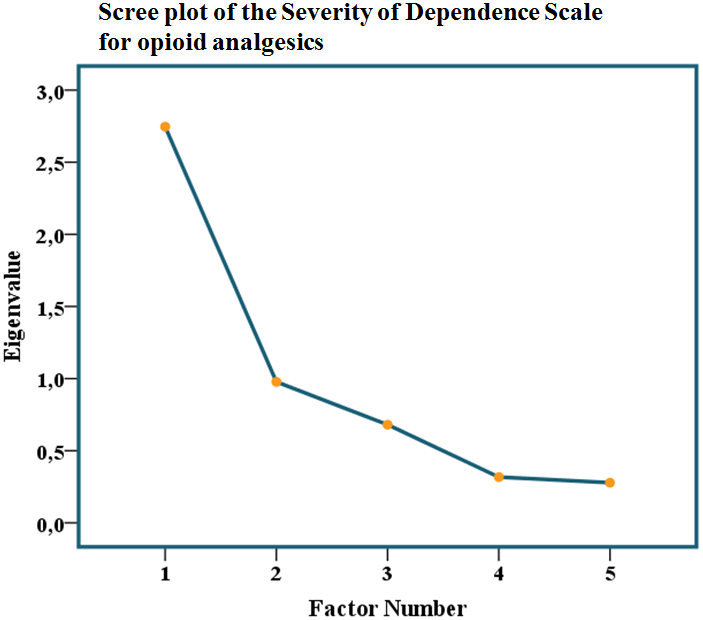

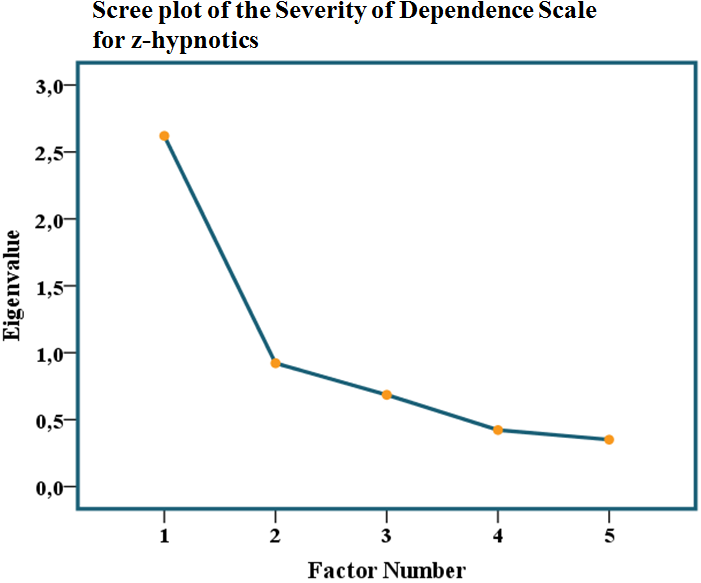

Supplement: Supplementary file 3 — Scree plots of the Severity of Dependence Scale for each group of medications. (DOCX 189 kb) [file 12877_2019_1182_MOESM3_ESM.docx]
