# Supplementary material for: The Severity of Dependence Scale detects medication misuse and dependence among hospitalized older patients
Source: BMC Geriatr. 2019 Jun 24;19:174. doi: 10.1186/s12877-019-1182-3 (PMC6591833; doi:10.1186/s12877-019-1182-3)
Supplement: Supplementary file 2 — Cutoff values and accuracy indices of the Severity of Dependence Scale for benzodiazepines, opioid analgesics and z-hypnotics. (DOCX 15 kb) [file 12877_2019_1182_MOESM2_ESM.docx]

**Additional file 2** Cutoff values and accuracy indices of the Severity of Dependence Scale for benzodiazepines, opioid analgesics and z-hypnotics

| **Medication** | **Optimal cutoff** | **Sensitivity** | **Specificity** | **PPV** | **NPV** | **LR+** | **LR-** | **χ2 test**  **(*P*-value)** | **Youden's index** | **Euclidean's distance** |
| --- | --- | --- | --- | --- | --- | --- | --- | --- | --- | --- |
| Benzodiazepine | 5.5 | 0.63 | 1.00 | 1.00 | 0.80 | NA | 0.38 | 10.00 (<0.05) | 0.63 | 0.38 |
| Opioid analgesics | 4.5 | 0.93 | 0.77 | 0.65 | 0.96 | 4.11 | 0.09 | 19.29 (<0.001) | 0.70 | 0.23 |
| Z-hypnotics | 5.5 | 0.75 | 0.85 | 0.78 | 0.83 | 5.00 | 0.29 | 24.77 (<0.001) | 0.60 | 0.29 |
