# Supplementary material for: The Severity of Dependence Scale detects medication misuse and dependence among hospitalized older patients
Source: BMC Geriatr. 2019 Jun 24;19:174. doi: 10.1186/s12877-019-1182-3 (PMC6591833; doi:10.1186/s12877-019-1182-3)
Supplement: Supplementary file 4 — Internal consistency and item loadings of the Severity of Dependence Scale for benzodiazepines. (DOCX 14 kb) [file 12877_2019_1182_MOESM4_ESM.docx]

**Additional file 4** Internal consistency and item loadings of the Severity of Dependence Scale for benzodiazepines

|  | **Internal consistency** | | | **Item loading** | |
| --- | --- | --- | --- | --- | --- |
|  | **Mean**  **(Scale variance)** | **Items-total correlation** | **Cronbach’s alpha if the item is eliminated** | **Factor 1** | **Factor 2** |
| 1. Do you think your use of benzodiazepine was out of control? | 3.45 (9.95) | -0.11 | 0.82 | -0.07 | 0.84 |
| 2. Did the prospect of missing a dose make you anxious or worried? | 2.85 (6.03) | 0.79 | 0.62 | 0.96 | -0.08 |
| 3. Did you worry about your use of benzodiazepine? | 2.90 (6.62) | 0.68 | 0.67 | 0.79 | -0.10 |
| 4. Did you wish you could stop? | 2.40 (6.04) | 0.46 | 0.75 | 0.55 | -0.12 |
| 5. How difficult would you find it to stop or go without using benzodiazepine? | 2.40 (4.78) | 0.73 | 0.63 | 0.88 | 0.38 |
