# Supplementary material for: The Severity of Dependence Scale detects medication misuse and dependence among hospitalized older patients
Source: BMC Geriatr. 2019 Jun 24;19:174. doi: 10.1186/s12877-019-1182-3 (PMC6591833; doi:10.1186/s12877-019-1182-3)
Supplement: Supplementary file 5 — Internal consistency and item loadings of the Severity of Dependence Scale for opioid analgesics. (DOCX 14 kb) [file 12877_2019_1182_MOESM5_ESM.docx]

**Additional file 5** Internal consistency and item loadings of the Severity of Dependence Scale for opioid analgesics

|  | **Internal consistency** | | | **Item loading** |
| --- | --- | --- | --- | --- |
|  | **Mean**  **(Scale variance)** | **Items-total correlation** | **Cronbach’s alpha if the item is eliminated** | **Factor 1** |
| 1. Do you think your use of opioid analgesics was out of control? | 3.93 (9.52) | 0.49 | 0.73 | 0.60 |
| 2. Did the prospect of missing a dose make you anxious or worried? | 3.49 (6.76) | 0.70 | 0.61 | 0.88 |
| 3. Did you worry about your use of opioid analgesics? | 3.60 (7.93) | 0.45 | 0.70 | 0.67 |
| 4. Did you wish you could stop? | 2.69 (6.04) | 0.49 | 0.72 | 0.56 |
| 5. How difficult would you find it to stop or go without using opioid analgesics? | 2.64 (6.51) | 0.56 | 0.66 | 0.60 |
