# Supplementary material for: The Severity of Dependence Scale detects medication misuse and dependence among hospitalized older patients
Source: BMC Geriatr. 2019 Jun 24;19:174. doi: 10.1186/s12877-019-1182-3 (PMC6591833; doi:10.1186/s12877-019-1182-3)
Supplement: Supplementary file 6 — Internal consistency and item loadings of the Severity of Dependence Scale for z-hypnotics. (DOCX 17 kb) [file 12877_2019_1182_MOESM6_ESM.docx]

**Additional file 6** Internal consistency and item loadings of the Severity of Dependence Scale for z-hypnotics

|  | **Internal consistency** | | | **Item loading** |
| --- | --- | --- | --- | --- |
|  | **Mean**  **(Scale variance)** | **Items-total correlation** | **Cronbach’s alpha if the item is eliminated** | **Factor 1** |
| 1. Do you think your use of z-hypnotics was out of control? | 4.21 (9.09) | 0.44 | 0.70 | 0.51 |
| 2. Did the prospect of missing a dose make you anxious or worried? | 3.57 (6.67) | 0.66 | 0.59 | 0.82 |
| 3. Did you worry about your use of z-hypnotics? | 3.79 (7.27) | 0.60 | 0.62 | 0.74 |
| 4. Did you wish you could stop? | 3.04 (7.71) | 0.25 | 0.78 | 0.29 |
| 5. How difficult would you find it to stop or go without using z-hypnotics? | 3.09 (6.35) | 0.56 | 0.63 | 0.76 |
