# Supplementary material for: The Severity of Dependence Scale detects medication misuse and dependence among hospitalized older patients
Source: BMC Geriatr. 2019 Jun 24;19:174. doi: 10.1186/s12877-019-1182-3 (PMC6591833; doi:10.1186/s12877-019-1182-3)
Supplement: Supplementary file 7 — Severity of dependence scale (SDS). (DOCX 14 kb) [file 12877_2019_1182_MOESM7_ESM.docx]

**Additional file 7: SEVERITY OF DEPENDENCE SCALE (SDS)**

| 1. Do you think your use of ….. (drug) was out of control? | 🞏 0. Never/almost never | 🞏1. Sometimes | 🞏 2. Often | 🞏 3. Always |
| --- | --- | --- | --- | --- |
| 2. Did the prospect of missing a dose make you anxious or worried? | 🞏 0. Never/almost never | 🞏1. Sometimes | 🞏 2. Often | 🞏 3. Always |
| 3. Did you worry about your use of the drug? | 🞏 0. Never/almost never | 🞏1. Sometimes | 🞏 2. Often | 🞏 3. Always |
| 4. Did you wish you could stop? | 🞏 0. Never/almost never | 🞏1. Sometimes | 🞏 2. Often | 🞏 3. Always |
| 5. How difficult would you find it to stop or go without ….. (drug)? | 🞏 0. Not difficult | 🞏1. Quite difficult | 🞏 2. Very difficult | 🞏 3. Impossible |

TOTAL SCORE____________
