# Supplementary material for: The Severity of Dependence Scale detects medication misuse and dependence among hospitalized older patients
Source: BMC Geriatr. 2019 Jun 24;19:174. doi: 10.1186/s12877-019-1182-3 (PMC6591833; doi:10.1186/s12877-019-1182-3)
Supplement: Supplementary file 8 — Mini international neuropsychiatric interview (DSM-IV criteria, Version 6.0.0). (DOCX 26 kb) [file 12877_2019_1182_MOESM8_ESM.docx]

**Additional file 8: MINI INTERNATIONAL NEUROPSYCHIATRIC INTERVIEW**

**DSM-IV criteria, Version 6.0.0**

**PSYCHOACTIVE SUBSTANCE USE DISORDERS (NON-ALCOHOL)**

**( means: go to the diagnostic boxes, circle NO in all diagnostic boxes, and move to the next module)**

**Now I am going to show you/read to you a list of street drugs or medicines.**

**J1** **Have you in the past 12 months ever taken** any of these drugs more than once to get high, NO YES

to feel better, or to change your mood?

**CIRCLE EACH DRUG TAKEN:**

**Stimulants:** amphetamines, "speed", methamphetamine (crystal meth), “crank”, “rush”, Dexedrine, Ritalin, diet pills.

**Cocaine:** cocaine, snorting, IV, freebase, crack, "speedball".

**Opiates:** heroin, morphine, opium, methadone, codeine, OxyContin.

**Hallucinogens:** LSD ("acid"), mescaline, peyote, psilocybin, STP, "mushrooms", “ecstasy”, MDA, MDMA.

**Phencyclidin:** PCP ("Angel Dust", "PeaCe Pill", “Tranq”) or ketamin (”special K”).

**Inhalants:** glue, ethyl chloride, "rush", dinitrogen monoxide ("laughing gas"), amyl or butyl nitrate ("poppers").

**Cannabis:** marijuana, hashish ("hash"), THC, "reefer", "grass".

**Anxiolytics:** Valium, Vival, Stesolid, Xanor (alprazolam), Seconal, Librium, Ativan, Halcion, barbiturates, GHB, Rohypnol (“Roofies”).

**Miscellaneous:** steroids, nonprescription sleep or diet pills, cough syrup. Any others?

SPECIFY MOST USED DRUG(S):

WHICH SUBSTANCE(S)/MEDICATION(S) CAUSE THE MAJOR PROBLEMS?

**J2** **Considering your use of (name the drug / drug class selected), in the past 12 months:**

a. Have you found that you needed to use more (name of drug / drug class selected) NO YES

to get the same effect that you did when you first started taking it?

b. When you reduced or stopped using (name of drug / drug class selected), did you have NO YES

withdrawal symptoms (aches, shaking, fever, weakness, diarrhea, nausea, sweating,

heart pounding, difficulty sleeping, or feeling agitated, anxious, irritable, or depressed)?

Did you use any drug(s) to keep yourself from getting sick (withdrawal symptoms) or

so that you would feel better?

if **yes** to either question, code **yes**.

c. Have you often found that when you used (name of drug / drug class selected), NO YES

you ended up taking more than you thought you would?

d. Have you tried to reduce or stop taking (name of drug / drug class selected), but failed? NO YES

e. On the days that you used (name of drug / drug class selected), did you spend substantial NO YES

time (> 2 hours) in obtaining, using or in recovering from drug(s), or thinking about drug(s)?

f. Did you spend less time working, enjoying hobbies, or being with family or friends NO YES

because of your drug use?

g. Have you continued to use (name of drug / drug class selected) even though it caused NO YES

you health or mental problems?

| are **3** or more **J2** answers coded **yes**?  specify drug(s): **__________________________________**    * IF YES, SKIP J3 QUESTIONS | **NO YES**  ***SUBSTANCE DEPENDENCE***  **CURRENT** |
| --- | --- |

**J3** **Considering your use of (name the drug / drug class selected), in the past 12 months:**

a. Have you been intoxicated, high, or hungover from (name of drug / drug class selected) NO YES

more than once, when you had other responsibilities at school, at work, or at home?

Did this cause any problems?

(Code **yes** only if this caused problems.)

b. Have you been high or intoxicated from (name of drug / drug class selected) NO YES

more than once, in any situation where you were physically at risk, (for example,

driving a car, riding a motorbike, using machinery, boating, etc.)?

c. Did you have legal problems more than once, because of your drug use, NO YES

for example, an arrest or disorderly conduct?

d. Did you continue to use (name of drug / drug class selected) even though it caused NO YES

problems with your family or other people?

| are **1** or more **J3** answers coded yes?  specify drug(s): __________________________________ | **NO YES**  ***SUBSTANCE ABUSE***  **CURRENT** |
| --- | --- |
